# Supplementary material for: Association of 100% Fruit Juice Consumption with Cognitive Measures, Anxiety, and Depression in US Adults
Source: Nutrients. 2022 Nov 15;14(22):4827. doi: 10.3390/nu14224827 (PMC9696008; doi:10.3390/nu14224827)
Supplement: Supplementary file 1 [file nutrients-14-04827-s001.zip › nutrients-2005849-supplementary.pdf]

Association of 100% fruit juice consumption with cognitive measures, anxiety, and depression in US adults.

Supplemental Table S1. Association of various additional covariates on neurocognitive outcomes in NHANES[1].

Age (Years)	Neurocognitive Outcome	Covariates													
		HEI-2015 score	Weekday Sleep Hours	Anti-Dep. Med	Hyper tension Med	Edu. Level	Glyco-Hgb	Elevated BP	Doctor Told Stroke	Caffeine	Vit B12	Iron	Folic Acid	Whole fruits	Total Vege-tables
20+	Feel Anxious	NS	NS	POS	NS	INV	NS	NS	POS	POS	NS	NS	NS	NS	NS
	Feeling Depressed Level	NS	INV	POS	NS	INV	NS	NS	POS	NS	NS	NS	NS	INV	INV
20-59	Feel Anxious	NS	NS	POS	NS	NS	NS	NS	NS	POS	NS	NS	NS	INV	NS
	Feeling Depressed Level	NS	INV	POS	NS	INV	NS	NS	POS	NS	NS	NS	NS	INV	INV
	Simple Reaction Time Mean	NA	NA	NS	NS	INV	NS	NS	NS	NS	NS	NS	NS	NS	NS
	Single Digit Learning Total Score	NA	NA	NS	NS	INV	POS	NS	NS	INV	NS	INV	INV	NS	INV
	Symbol Digital Substitution Mean	NA	NA	NS	POS	INV	NS	NS	NS	INV	NS	INV	INV	NS	INV
60+	Animal Fluency Score	NS	NS	NS	NS	POS	NS	NS	NS	NS	POS	NS	NS	NS	POS
	CERAD: Score Delayed Recall	NS	INV	INV	NS	POS	INV	NS	NS	NS	NS	NS	NS	NS	NS
	CERAD: Total Score Recall	NS	NS	INV	NS	POS	NS	INV	NS	NS	NS	NS	NS	NS	POS
	Digital Symbol Score	NS	INV	INV	NS	POS	INV	NS	INV	NS	POS	NS	NS	NS	NS
	Feel Anxious	INV	INV	POS	NS	NS	NS	NS	POS	NS	NS	NS	INV	POS	NS
	Feeling Depressed Level	NS	INV	POS	NS	INV	NS	NS	POS	NS	NS	NS	NS	NS	INV
	Trouble Remembering	NS	POS	NS	NS	NS	NS	NS	NS	NS	NS	NS	NS	NS	NS

[1]Data from NHANES 1988-1994, 1999-2018; Results from regression analyses of age, gender, ethnicity, physical activity level, poverty income ratio level, weight status, current smoking status, Healthy Eating Index 2015, weekday hours of sleep, antidepressant medication use, hypertension medication use, education level, glycohemoglobin, elevated blood pressure, and doctor told you had a stroke, and intakes of caffeine, vitamin B12, iron, folic acid, total fruits, whole fruits, and total vegetables excluding legumes; NS: not significant; POS: positive regression coefficient, p<0.05; INV: negative regression coefficient. p<.05; NA: not available.
